# Hydrogen peroxide in breast milk is crucial for gut microbiota formation and myelin development in neonatal mice

**DOI:** 10.1080/19490976.2024.2359729

**Published:** 2024-05-30

**Authors:** Jun Kambe, Kento Usuda, Ryo Inoue, Kazuhiko Hirayama, Masahiko Ito, Ken Suenaga, Sora Masukado, Hong Liu, Shiho Miyata, Chunmei Li, Ikuo Kimura, Yuki Yamamoto, Kentaro Nagaoka

**Affiliations:** a Laboratory of Veterinary Physiology, Cooperative Department of Veterinary Medicine, Tokyo University of Agriculture and Technology, Tokyo, Japan; b Laboratory of Animal Science, Department of Applied Biological Sciences, Faculty of Agriculture, Setsunan University, Osaka, Japan; c Laboratory of Veterinary Public Health, Department of Veterinary Medical Science, Graduate School of Agricultural and Life Sciences, University of Tokyo, Tokyo, Japan; d Department of Virology and Parasitology, Hamamatsu University School of Medicine, Shizuoka, Japan; e College of Animal Science and Technology, Nanjing Agricultural University, Nanjing, China; f Laboratory of Molecular Neurobiology, Graduate School of Biostudies, Kyoto University, Kyoto, Japan

**Keywords:** Gut-brain axis, brain development, myelin, metabolites, behavior, microbiome

## Abstract

Early life environment influences mammalian brain development, a growing area of research within the Developmental Origins of Health and Disease framework, necessitating a deeper understanding of early life factors on children’s brain development. This study introduces a mouse model, *LAO1* knockout mice, to investigate the relationship between breast milk, the gut microbiome, and brain development. The results reveal that breast milk‘s reactive oxygen species (ROS) are vital in shaping the neonatal gut microbiota. Decreased hydrogen peroxide (H_2_O_2_) levels in milk disrupt the gut microbiome and lead to abnormal metabolite production, including D-glucaric acid. This metabolite inhibits hippocampal myelin formation during infancy, potentially contributing to behavioral abnormalities observed in adulthood. These findings suggest that H_2_O_2_ in breast milk is crucial for normal gut microbiota formation and brain development, with implications for understanding and potentially treating neurodevelopmental disorders in humans.

## 1. Introduction

Brain development in mammals is known to be significantly influenced by the external environment, particularly during early life. The early life environment has recently garnered substantial attention in studying neurodevelopmental disorders, such as autism spectrum disorder (ASD), attention deficit hyperactivity disorder (ADHD), and anxiety. The Developmental Origins of Health and Disease (DOHaD) framework posits that the early life environment profoundly impacts health, gaining significant traction.^1^ Hence, understanding the effects of inputs at early life stages on children’s brain development and the relationship between these factors and neurodevelopmental disorders is necessary. Several factors, including nutritional exposure, stress, and medication history during fetal and postnatal periods, may affect offspring brain development^2^; however, precise mechanisms that influence brain development remain elusive.

Recently, the influence of gut microbiomes on neurodevelopmental disorders has gained attention. The connection between the gut microbiome and conditions such as ASD and ADHD has been reported in patient studies and mouse models.^3^ Various pathways, including bacterial metabolites, the immune system, the vagus nerve system, and hormonal signaling, have been implicated in mediating changes in the gut microbiome and neurodevelopmental disorders.^4^ Specific metabolites associated with the gut microbiome have been identified in the blood of patients with neurodevelopmental abnormalities, including heightened anxiety and diminished social behavior; mouse studies have demonstrated that administering these metabolites induces behavioral abnormalities similar to those observed in patients,^5–7^ Based on gut microbiota diversity and several reported associations between the microbiome and neurodevelopmental disorders, many other gut microbiota metabolites are assumed to be involved in these diseases.

Breast milk has been highlighted as pivotal for brain development, with evidence suggesting that its intake enhances white matter volume and connectivity between brain regions.^8,9^ It is integral in establishing the neonatal gut microbiome. Neonates exclusively fed breast milk have been found to possess a higher abundance of lactic acid bacteria, including *Bifidobacterium* and *Lactobacillus*, in their gut microbiome than those raised solely on artificial milk.^10^ Moreover, breastfed neonates have a healthy and youthful microbiome.^11^ These studies imply a complex interplay between breast milk, brain development, and gut microbiome formation. However, specific constituents of breast milk that regulate gut microbiome composition and brain development remain unclear, primarily because a suitable animal model is absent to investigate this communication.

Reactive oxygen species (ROS) have been acknowledged for their antibacterial properties and use as enzymatic catalysts. They serve as defense mechanisms in mammals and bacteria against external organisms.^12^ Breast milk contains several enzymes that produce hydrogen peroxide (H_2_O_2_), a molecule released by cells and nearby bacteria. One such enzyme is L-amino acid oxidase (LAO), a flavoenzyme that catalyzes L-amino acid oxidation to generate keto acids, ammonia, and H_2_O_2_. Most mammals possess two related proteins encoded by LAO1 and interleukin 4-induced gene 1 (IL4I1), which are expressed in lactating mammary glands and immune system cells, respectively.^13,14^ Mouse milk contains abundant LAO1 levels, and its depletion alters the gut microbiota composition of lactating neonates.^15^ *LAO1* knockout (KO) adult mice show learning and memory failures owing to impairments in amino acid metabolism in the brain.^16^ Furthermore, a recent study has suggested that gut-derived LAO1 influences the metabolomic profile in the blood, with potential implications for hepatic function^17^; these findings imply that LAO1 may exert effects on the metabolome at multiple sites. Our preliminary studies using *LAO1* KO mice suggest that milk differences during lactation are associated with behavioral changes after growth; thus, we propose that *LAO1* KO mice are optimal models to investigate the relationship linking breast milk, gut microbiome formation, and brain development.

Here, we report a potential mechanism by which breast milk affects neonatal brain development via the gut microbiota, which is related to brain function after growth. We conducted behavioral tests using *LAO1* KO adult mice, utilizing microarray for gene expression profiling in the neonatal hippocampus, immunofluorescence for myelin-related proteins, fecal microbiome transplantation, microbiome profiling, fecal and serum metabolome, candidate metabolite administration to mice, and *in vitro* primary oligodendrocyte precursor cell culture. We found that H_2_O_2_ in breast milk promotes neonatal gut microbiome development and is critical for brain development; decreased H_2_O_2_ levels disrupt the microbiome and cause abnormal metabolite production during infancy. D-glucaric acid metabolite may inhibit myelin formation in hippocampal oligodendrocytes (OLs) and is implicated in behavioral abnormalities after growth.

## Material and methods

### Animals

All experiments involving mice were conducted according to ethical guidelines and approved by the University Animal Care and Use Committee of Tokyo University of Agriculture and Technology (approval codes: R03–110, R04–108) and The University of Tokyo (approval number: P15–132). *LAO1* KO mice were generated as previously described.^13^ WT and *LAO1* KO mice with a C57BL/6Jcl genetic background were maintained at 23 ± 2°C under a 14-h light cycle (lights on from 05:00 to 19:00). The mice had *ad libitum* access to chow (MR Breeder; Nosan Corporation, Kanagawa, Japan) and tap water. Adult WT and *LAO1* KO females were mated with male mice, and litter sizes were standardized to 6 ± 2 neonates. For cross-fostering, neonates were switched on P0 within 16 h (Supplementary Figure S1). For neonates (P10) and adults (P70), cecal feces, serum, stomach content, ileum, rectum, liver, and hippocampal samples were collected and stored at −80°C until further analysis. Neonatal mice included both sexes, whereas adult mice consisted of males only. Weaning occurred on P21, and mice with identical genotypes were cohoused until behavioral testing.

### Fecal transplantation

GF BALB/cA male mice were maintained at 24 ± 1°C under a 12-h lighting schedule with *ad libitum* access to food and water at The University of Tokyo. Cecal feces from P10 mice were orally administered to GF mice. Cecal feces were collected from P10 mice under anesthesia and stored in an AnaeroPack-carbon dioxide (CO_2_) environment (Mitsubishi Gas Chemical Company Inc., Tokyo, Japan) until transplantation. Each feces was diluted 100 times (w/v) using trypticase soy broth without dextrose (pH 7.2), which was anaerobic, and 0.84 g/L of sodium carbonate, 0.5 g/L Bacto Agar, and 0.5 g/L L-Cysteine was added. Diluted solutions were administered to four-week-old GF mice (0.5 mL) via oral gavage using a feeding needle. After two and six weeks, cecal feces, serum, and hippocampal samples were collected and preserved at −80°C for subsequent analysis.

### D-glucaric acid administration

Adult female WT mice were mated with adult male WT mice, and litter sizes were standardized to 8 ± 2 neonates. Littermates were randomly divided into two groups and orally administered saline or D-glucaric acid (100 mg/kg/day) from P1 until P10.

Eight-week-old WT male mice were randomly assigned to control and D-glucaric acid treatment groups. D-glucaric acid (0.5 g/L) was administered orally in their drinking water for forty-seven days.

Tissue samples for GC-MS-based metabolomics were obtained two hours after D-glucaric acid (100 mg/kg) was administered to either P10 or eight-week-old WT male mice. To account for blood contamination in the tissue, the mice were perfused with ice-cold 1 mM MOPS (pH 7.0) under anesthesia to eliminate blood from the tissue prior to sampling.

### Hydrogen peroxide (H_2_O_2_) administration

Adult female *LAO1* KO mice were mated with adult male *LAO1* KO mice, and litter sizes were standardized to 5 ± 3 neonates. A previous study indicated a shared gut microbiome among littermates even when treated^18,19^; hence, littermates were excluded from this experiment. Each dam was randomly assigned as saline or H_2_O_2_ (1 μg/day to 50 μg/day) group on P1, and the neonate was administered half the H_2_O_2_ orally in the morning and the remaining in the evening until P10. Fecal, serum, rectum, ileum, and hippocampal samples were collected from P10 neonates.

### Behavioral test

Before commencing behavioral testing, mice were handled by the experimenter for 1 min daily over three consecutive days. Behavioral tests were recorded and analyzed using the AnyMaze automated tracking system (Muromachi Kikai Co., Ltd., Tokyo, Japan).

#### Open field test

The open-field test was conducted as previously described, with some modifications.^20^ A square cage measuring 40 × 40 × 30 cm was used. The floor center was illuminated to 100 lux. Each mouse was placed in an open-field apparatus and recorded for 10 min. The total distance traveled, time spent in the center area (20 × 20 cm), number of entries into the center area, and average speed were assessed.

#### Morris water maze

The Morris water maze test was conducted as previously described.^21^ A circular polyethylene pool (100 cm in diameter) was filled with tap water to a depth of 50 cm, and nontoxic white paint was added to make the water opaque. A transparent circular escape platform (10 cm in diameter) was placed at a fixed location in the northeast quadrant and submerged 1 cm below the water surface. Four distinct visual cues were presented around the maze perimeter. Mice were placed in the southeast, south, southwest, west, or northwest positions and allowed to explore the pool for 60 s. If the mouse reached the escape platform within 60 s, it remained on it for 30 s; on failing to reach the platform within the designated time, the researcher guided them to it and allowed them to stay there for 30 s to memorize their position. The mice underwent this process four times daily for six consecutive days (training period). After a 24-h interval following training, a probe test was conducted. The mice explored the pool for 60 s without the escape platform and time spent swimming in the northeast quadrant (initial location of the escape platform) was measured.

#### Object location test

The object location test was performed as previously described with slightly modified.^22,23^ The test apparatus was the same as the OFT, with the center of the floor illuminated to a brightness of 45 lux. During the initial three days, mice were placed in the corner of the apparatus, allowing them to familiarize themselves with the environment for 10 min with no object. The training session was performed on the fourth day, placing two identical objects centrally 10 cm apart on the side wall. Mice freely explored the apparatus for 10 min as a training session. On the subsequent fifth day, one object was placed in the same place as on day four (familiar object), while another object was placed in a new location (moved object) (Supplementary Figure S2A). The test session was 10 min, during which the mice were allowed to explore the apparatus. The discrimination index was calculated as ((investigation time of moved object – investigation time of familiar object)/total investigation time of moved and familiar object) × 100. ANY-maze measured the investigation time of the moved object and the familiar object.

### RNA analysis

Total RNA was extracted from the hippocampus, liver, ileum, and rectum using ISOGEN II Reagent (Nippon Gene Co., Ltd., Toyama, Japan) as per the manufacturer’s instructions. RNA quantity was determined using a NanoDrop instrument (Thermo Fisher Scientific, MA, USA). For microarray analysis using RNA from the hippocampus, 20 μg of total RNA from each sample was utilized for Clariom™ S Assay (mouse, Thermo Fisher Scientific). Synthesis of cDNA was performed using PrimeScript™ II 1st strand cDNA Synthesis Kit (Takara Bio Inc., Shiga, Japan). For qRT-PCR, oligonucleotide primers were designed using Primer-BLAST (NCBI), and their sequences are presented in Supplementary Table S1. PCR reactions were performed in a 10 μl volume using PowerUp™ SYBR™ Green Master Mix (Thermo Fisher Scientific) and 7500 Fast Real-Time PCR system (Thermo Fisher Scientific), as per manufacturer’s instructions. Tubulin was used as an internal standard for each sample. Data were analyzed using the relative quantity (^ΔΔ^Ct) method.

### DNA extraction from cecal feces samples

DNA was isolated from cecal feces using QuickGene DNA Tissue Kit S (Kurabo Industries, Ltd., Osaka, Japan), as per manufacturer’s instructions. Briefly, 50 mg of fecal samples and 250 µl of tissue lysis buffer (MDT) were placed in a 2.0 ml tube and 15 mg of 0.1 mm φ glass beads were added. Mechanical homogenization was performed twice at 3000 rpm for 2 min using a homogenizer. Subsequently, 25 µl proteinase K (EDT) was added to each tube, followed by incubation at 55°C for 60 min. After incubation, samples were centrifuged at 15,000 ×g for 10 min at 16°C; 200 µl supernatant was collected and mixed with 180 µl lysis buffer (LDT). The solution was heated at 70°C for 10 min and 240 µl of 99.5% ethanol was added. QuickGene-Mini80 (Kurabo Industries, Ltd.) was used to purify crude DNA lysate as per manufacturer’s instructions. DNA concentration and quality was assessed using a NanoDrop spectrophotometer (Thermo Fisher Scientific). Samples were stored at −20°C for subsequent analysis.

### Library preparation and DNA sequencing

Fecal samples were subjected to 16S rRNA metagenome analysis as previously described.^24^ Briefly, the first PCR targeted variable regions 3 and 4 (V3–4) of the 16S rRNA gene using primers 341F (CCTACGGGNGGCWGCAG) and 805 R (GACTACHVGGGTATCTAATCC), followed by a second PCR to attach dual indices. Amplicons were pooled equally and a library of 10 pM concentration was combined with the phiX control and sequenced using a MiSeq v3 kit as per manufacturer’s instructions. Data processing, including chimera checking, amplicon sequence variant (ASV) definition, and taxonomic assignment, was performed using DADA2, and QIIME 2 2021.2 was used for denoising and singleton deletion.^25^ A reference from Silva 138 was used for the taxonomic assignment. Microbiome-based prediction of enzyme expression was performed using PICRUSt2,^26–31^ MicrobiomeAnalyst 2.0 was used for statistical analysis, with data filtering steps, including a minimum count of 2 and 10% prevalence in samples.^32^ Default settings were used for all other steps except those mentioned.

### Perfusion and immunofluorescent analysis

On experiment completion, mice were deeply anesthetized using a mixture of three anesthetic agents (0.3 mg/kg medetomidine, 4.0 mg/kg midazolam, and 5.0 mg/kg butorphanol, intraperitoneal injection) combined with isoflurane inhalation. Subsequently, perfusion was performed transcardially using phosphate-buffered saline (PBS), followed by 4% paraformaldehyde in PBS at 0.1 mL per second. Post-perfusion, the brain was removed, post-fixed in 4% paraformaldehyde overnight at 4°C, cryoprotected in a 30% sucrose in PBS solution at 4°C for 24–72 h, and embedded in optimal cutting temperature compound (Tissue-Tek, Sakura Finetek Japan, Tokyo, Japan). Brain sections of 25 µm thickness were obtained using a cryostat and placed on MAS-coated glass slides (Matsunami Glass, Osaka, Japan). Sections with slides were washed thrice with PBS, then blocked in PBS containing 5% bovine serum albumin (BSA, Sigma-Aldrich, MO, USA) and 0.3% Triton X-100 for 2 h at 16°C. After blocking, sections were incubated overnight at 4°C in PBS containing 1% BSA, 0.3% Triton X-100, rabbit monoclonal anti-PLP1 primary antibody (dilution: 1:200; #28702 Cell Signaling Technology, MA, USA), or rabbit monoclonal anti-MBP primary antibody (dilution: 1:500, #78896 Cell Signaling Technology). Subsequently, sections were washed three times with PBS, followed by incubation in PBS containing 1% BSA, 0.3% Triton X-100, and secondary goat anti-rabbit Alexa Fluor 647 antibody (dilution: 1:1000, Thermo Fisher Scientific, A-21244). Sections were then washed with PBS and mounted with DAPI-containing mounting medium under a cover glass. The MBP and PLP1-positive area of the dorsal hippocampus was acquired from the stratum oriens of CA1 to the molecular layer of dentate gyrus via Fiji, using three sections from each animal.

### OPC proliferation and differentiation assay

OPC were purified following a previously described method, with slight modifications.^33,34^ Briefly, whole brains were dissected from P1 mice and only the cortices were retained after extraction under a stereomicroscope. Cortical tissue was diced into pieces smaller than 1 mm^3^ using spring scissors in a 100 mm dish (Corning Inc., NY, USA). The minced cortical tissue was then mechanically triturated in DMEM/F-12 (Thermo Fisher Scientific) supplemented with 20% fetal bovine serum (Hyclone Laboratories Inc., D.C., USA), 1× antibiotic antimycotic solution (Merck, NJ, USA), and 0.01 M HEPES (Thermo Fisher Scientific). Cortical tissue from a single mouse was transferred into a *T*-25 flask (Corning) and incubated in a 5% CO2 environment at 37°C. The medium was replaced after 84 h, followed by medium replacement every other day. OPCs were shaken off when the mixed glial cultures almost covered the surface. The cell suspension was collected and filtered through a Falcon® 40 µm Cell Strainer (Corning) to remove small astrocyte clumps. The collected cell pellet was resuspended in OPC differentiation medium consisting of DMEM/F12 (Thermo Fisher Scientific) supplemented with 1× N2 (Thermo Fisher Scientific), 1× B27 (Thermo Fisher Scientific), 1× antibiotic antimycotic solution (Merck), 0.1% w/v BSA (Merck), and 10 ng/ml triiodothyronine (Merck). Cell suspensions were transferred to untreated Petri dishes (Corning) and incubated for 30 min in a 5% CO2 environment at 37°C. OPCs suspended in this manner were collected and quantified using a hemocytometer. OPCs were plated at 1 × 10^3^ cells/cm^2^ density onto Poly-D-lysine (PDL; Thermo Fisher Scientific, 50 μg/mL) and Laminin (LN; Sigma, 10 μg/mL) coated plates (Corning). This protocol consistently yielded 70–80% OPC purity. Three days after plating, cells were used for transcription analysis. Total RNA was isolated using ISOGEN II Reagent (Nippon Gene Co., Ltd.) as per the manufacturer’s instructions.

### GC-MS based non-targeted metabolomics

Non-targeted metabolomics was conducted using gas chromatography-mass spectrometry (GC-MS), with some modifications.^16^ Briefly, cecal feces was diluted five-fold (weight/volume) with ultrapure water (Thermo Fisher Scientific). The supernatant was collected after centrifugation at 20,000 ×g at 4°C for 5 min. Serum was diluted five-fold with ultrapure water. For breast milk samples, five times the volume of ultrapure water was added, followed by centrifugation at 21,500 ×g at 4°C for 60 min. Resultant intermediate layers were collected. Small debris was removed using a 0.22 μm filter. The subsequent steps were uniform for all samples. A total of 50 μl of filtered solutions were mixed with 1 mg/ml 2-isopropyl malic acid as an internal standard, along with 250 μl of methanol-ultra-pure water-chloroform (2.5:1:1). The mixture was incubated at 37°C for 30 min on a shaker. After incubation, samples were centrifuged at 16,000 ×g at 4°C for 5 min. The resulting supernatant (225 μl) was collected and mixed with 200 μl of ultra-pure water. This mixture was centrifuged again under the same conditions previously described. The supernatant (250 μl) was collected and vacuum-concentrated using a centrifuge evaporator for 20 min. Concentrated samples were frozen at −80°C and freeze-dried for 16 h. Freeze-dried samples were mixed with 40 μl of 20 mg/ml dehydrated pyridine and incubated at 37°C for 90 min on a shaker. Samples were then derivatized using 20 μl of N-methyl-N-trimethylsilyl trifluoroacetamide (MSTFA, Thermo Fisher Scientific) at 37°C for 45 min on a shaker. Finally, 1 μl of derivatized samples was used for GC-MS analysis (GCMS QP2020 NX, Shimadzu, Kyoto, Japan), following previously described protocol.

Tissue metabolomics was performed using GC-MS. In this procedure, 20 mg of tissues were combined with 1 mg/ml 2-isopropyl malic acid as an internal standard, along with 1000 μl of methanol-ultra-pure water-chloroform (2.5:1:1). The resulting mixture underwent homogenization and incubation at 37°C for 30 min on a shaker. After incubation, samples were centrifuged at 16,000 ×g at 4°C for 5 min. The 900 μl supernatant was mixed with 450 μl of chloroform, and then centrifugation was conducted under the same conditions. The 500 μl upper layer was collected, combined with 200 μl of ultra-pure water, and centrifuged with the same conditions. The 500 μl supernatant was vacuum-concentrated using a centrifuge evaporator for 60 min, followed by freezing at −80°C and freeze-dried for 16 h. The resulting freeze-dried samples were mixed with 80 μl of 20 mg/ml dehydrated pyridine and incubated at 37°C for 90 min on a shaker. Subsequently, samples were derivatized using 40 μl of MSTFA at 37°C for 30 min on a shaker. Finally, 1 μl of derivatized samples was injected into GC-MS.

Peaks were selected using Shimadzu Smart Metabolite database. Estimation of missing values, data filtering, normalization with 2-isopropylmalic acid, and auto-scaling of metabolomic data were performed using MetaboAnalyst 5.0 with default settings.^35^ Naïve partial least squares analysis was conducted using chemometrics packages in R^36,37^ and candidate metabolites were identified at a p-value threshold of less than 0.05. Venn diagrams were created using the VennDiagram package.

### Measurement of milk H_2_O_2_ concentration

Breast milk samples were collected from lactating dams on P10 via gentle suction under mixed anesthesia. Skim milk was prepared as described previously^15^ with slight modifications. In brief, 200 μl of breast milk was mixed with 1 ml of PBS, followed by centrifugation at 28,000 ×g at 4°C for 60 min. The middle layer was collected as skimmed milk. Protein concentration of skim milk was determined using Pierce™ Coomassie (Bradford) Protein Assay Kit (Thermo Fisher Scientific). Subsequently, 97 μl of skim milk samples (2 mg/ml) were incubated with 1 μl of 100× non-essential amino acids (NEAA; Merck) and 2 μl of 50× amino acids (AA; Merck) for 2 h. H_2_O_2_ concentration was assessed using Amplex™ Red Hydrogen Peroxide/Peroxidase Assay Kit (Thermo Fisher Scientific) with a Varioskan LUX instrument (Thermo Fisher Scientific).

### Statistical analyses

Mice were randomly divided into conditions. The behavioral experiments were conducted in a non-randomized and non-blind manner, but recorded data was analyzed using AnyMaze automated tracking system. Image analysis was performed by a blinded researcher. Data are presented as means ± standard error (SEM) with individual points also shown. Statistical analyses were performed using R version 3.5.0. Differences between the two groups were evaluated using an unpaired Student’s *t*-test. Sidak’s tests were performed to analyze the Morris water maze training period and body weight during D-glucaric acid administration. Dunnett’s test was used to analyze cell culture results. Mann-Whitney U test was used for metagenomic analysis. Statistical analysis method, *U*, *t*, *q*, and *p* values were presented in the figure legends. Statistical significance was set at p < 0.05.

Fold change and p-values related to the microarray data were computed using Transcriptome Analysis Console (TAC) 4.0 (Thermo Fisher Scientific). Furthermore, GO analysis was conducted using R Studio and clusterProfiler.^38^ The statistical significance of enriched GO terms was set at adjusted p (Q value) < 0.1 corrected by Benjamini-Hochberg.

## 2. Results

### Changes in breast milk compositions caused spatial memory impairment in adult mice

**Figure 1.**
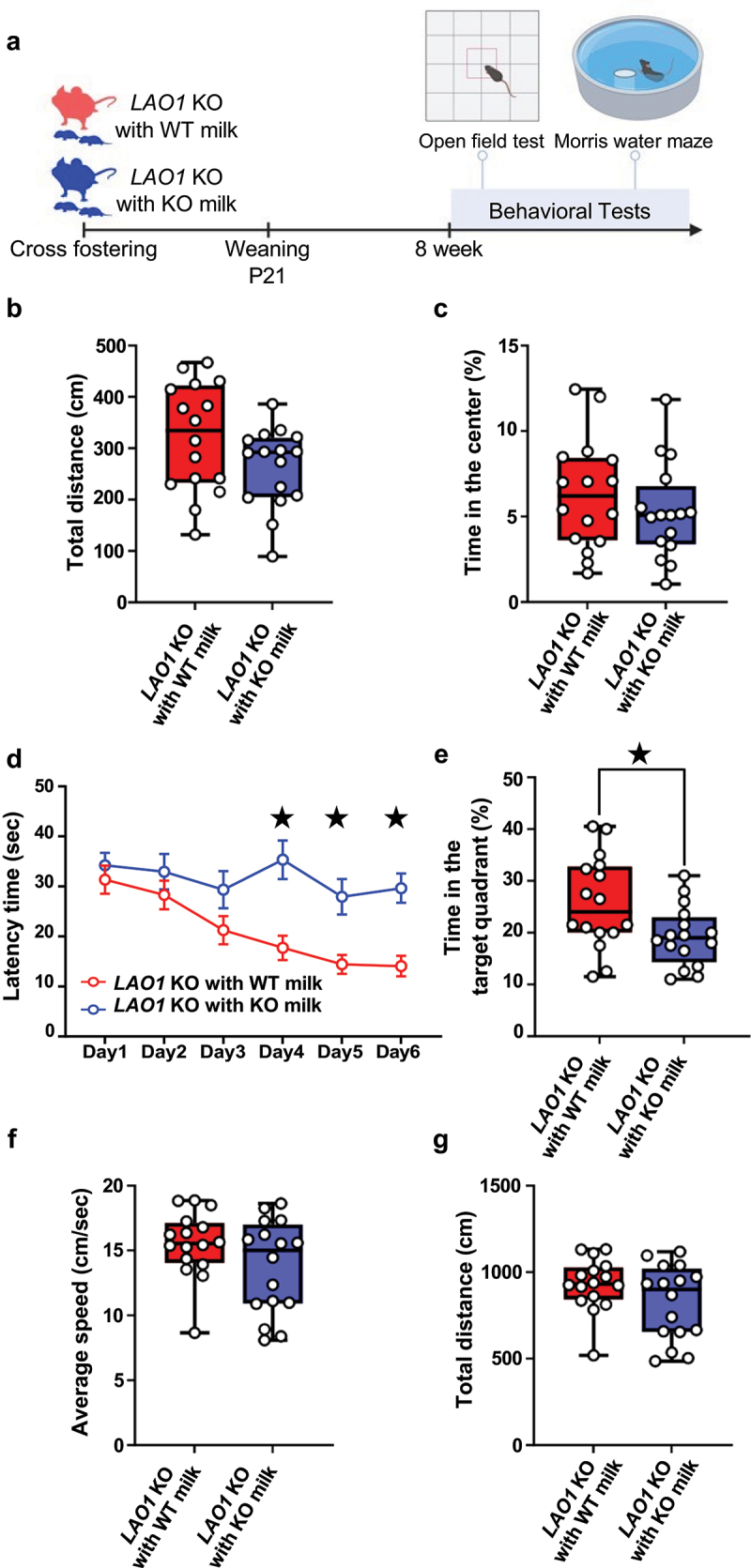
Changes in breast milk compositions caused spatial memory impairment in adult mice.

To eliminate the effects of LAO1 expressed in tissues other than the mother’s milk on gut microbiota formation and brain development, the offspring mice were genotyped as *LAO1* KO for cross-fostering (Supplementary Figure S1). First, we examined the effect of milk composition differences in *LAO1* KO mice on brain function after growth (Figure 1a). The open field test (OFT) results showed no impact on basic locomotion or anxiety-like behavior due to milk differences (Figures 1b,c). In the Morris water maze test, impaired spatial memory during training was observed in the *LAO1* KO with KO milk group (Figure 1d), further confirmed using the probe test (Figure 1e). No changes in locomotion or swimming ability were observed in the Morris water maze probe test (Figures 1f,g). Differences in responsiveness to stress might affect the results in the Morris water maze test because this test is dependent on aversion to water.^21,23^ Hence, we performed the object location test (OLT) on the *LAO1* KO with WT milk and *LAO1* KO with KO milk mice (Supplementary Figure S2A). The outcomes of the OLT revealed that the *LAO1* KO with KO milk group exhibited impairment of spatial memory, although the overall duration of object investigation remained unaltered (Supplementary Figures S2B and S2C).

(a) The changes in breast milk compositions influence on behavior was studied by behavior tests after cross fostering and the schematic diagram was illustrated. Created with BioRender.com.

(b) Locomotor activity in *LAO1* KO with WT milk and *LAO1* KO with KO milk as assessed in OFT. *n* = 16 each; *t_30_* = 1.787, *p* = 0.08, Student’s t-test.

(c) Anxiety-like behavior in *LAO1* KO with WT milk and *LAO1* KO with KO milk as assessed in OFT. *n* = 16 each; *t30* = 0.97, *p* = 0.34, Student’s t-test.

(d) Spatial learning in *LAO1* KO with WT milk and *LAO1* KO with KO milk as demonstrated using Morris water maze in training. Day1 (*n* = 16 each, *t_180_ = 0.68, p = 0.98*, Sidak’s tests), Day2 (*n* = 16 each, *t*_180_ = 1.01, *p* = 0.85, , Sidak’s tests), Day3 (*n* = 16 each, *t_180_*  = 1.92, *p* = 0.30, Sidak’s tests), Day4 (*n* = 16 each, *t*_180_ =  = 4.19, *p* < 0.05, Sidak’s tests), Day5 (*n* = 16 each, *t*_180_ = 3.21, *p* < 0.05, Sidak’s tests), Day6 (*n* = 16 each, *t_180_*  = 3.70, *p* < 0.05, Sidak’s tests).

(e) Spatial memory *LAO1* KO with WT milk and *LAO1* KO with KO milk as assessed in Morris water maze test in probe test. *n* = 16 each; *t*_30_ = = 2.42, *p* < 0.05, Student’s t-test.

(f and g) Swimming activity as assessed in Morris water maze test in probe test. F (*n* = 16 each, *t_30_* = 1.59, *p* = 0.12, Student’s *t*-test). G (*n* = 16 each, *t_30_* = 1.59, *p* = 0.12, Student’s *t*-test).

Mean ± SEM; ^★^indicates *p* <0.05; P, postnatal; KO, knockout; WT, wild-type.

### Changes in breast milk compositions suppressed myelin-related gene expression in neonatal mice

**Figure 2.**
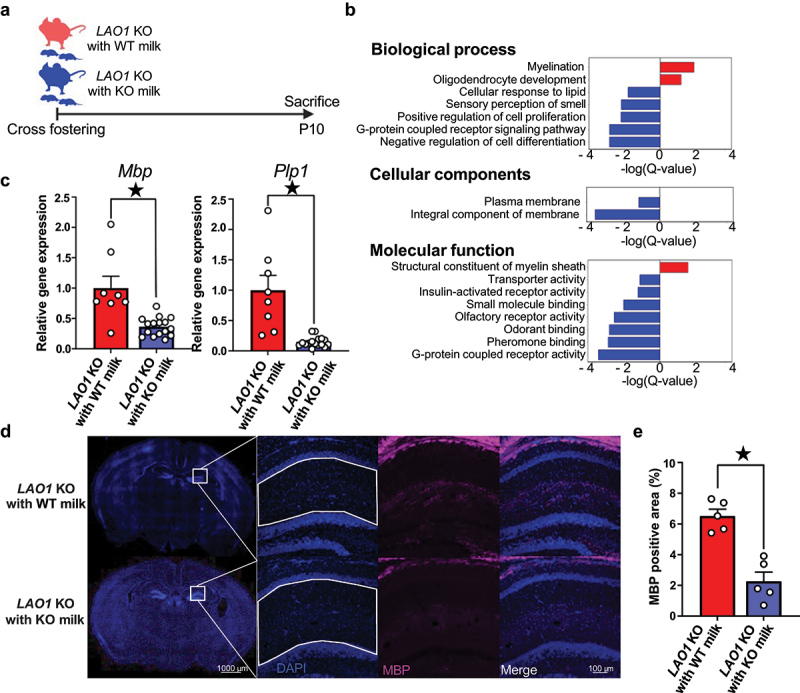
Changes in breast milk compositions suppressed myelin-related gene expression in neonatal mice.

Because the change in behavioral patterns after growth was thought to be the result of the influence of breast milk intake during infancy on the development of the hippocampus, we employed infant *LAO1* KO with wild-type (WT) milk and *LAO1* KO with KO milk (Figure 2a). We conducted a microarray analysis of postnatal day 10 (P10) hippocampal samples, which revealed altered myelin-related gene ontology (GO) terms (Figure 2b, Supplementary dataset1). Quantitative real-time polymerase chain reaction (qRT-PCR) confirmed reduced *Mbp* and *Plp1* expression in the *LAO1* KO with KO milk group compared to that in *LAO1* KO with WT milk (Figure 2c). Immunostaining indicated reduced myelin basic protein (MBP) levels in the hippocampus on P10 (Figure 2d). Quantifying the MBP-positive area in the dorsal hippocampal region showed a significant decrease in the *LAO1* KO with KO milk group (Figure 2e). In summary, LAO1-deficient breastfed mice showed reduced myelin formation in the hippocampus during infancy and impaired spatial memory after growth.

(a) The changes in breast milk compositions influence on brain development was studied after cross fostering and the experimental flow was illustrated. Created with BioRender.com.

(b) Functional enrichment analysis using microarray results revealed significantly enriched gene ontology (GO) terms; the red bar indicates up-regulated GO terms in *LAO1* KO with WT milk, and the blue bar indicates downregulated GO terms in *LAO1* KO with WT milk. *n* = 3 each, threshold of significance: q-value <0.1, Benjamini-Hochberg correction.

(c) Quantitative real-time polymerase chain reaction (qRT-PCR) validation of microarray data of *Mbp* and *Plp1* using P10 hippocampus. *Mbp* (*LAO1* KO with WT milk *n* = 8, *LAO1* KO with KO milk *n* = 16, *t*_*22*_ = 4.33, *p* < 0.05, Student’s *t*-test), *Plp1* (*LAO1* KO with WT milk *n* = 8, *LAO1* KO with KO milk *n* = 16, *t*_*22*_ = 5.02, *p* < 0.05, Student’s *t*-test).

(d and e) Representative myelin basic protein (MBP) staining and quantitative analysis at P10 dorsal hippocampus. *n* = 5 each, *t*_*8*_ = 5.73, *p* < 0.05, Student’s *t*-test.

Mean ± SEM; ^★^ indicates *p* < 0.05; P, postnatal; KO, knockout; WT, wild-type.

### Fecal transplants from different breastfed neonates affect myelin-related gene expression in germ-free mice

**Figure 3.**
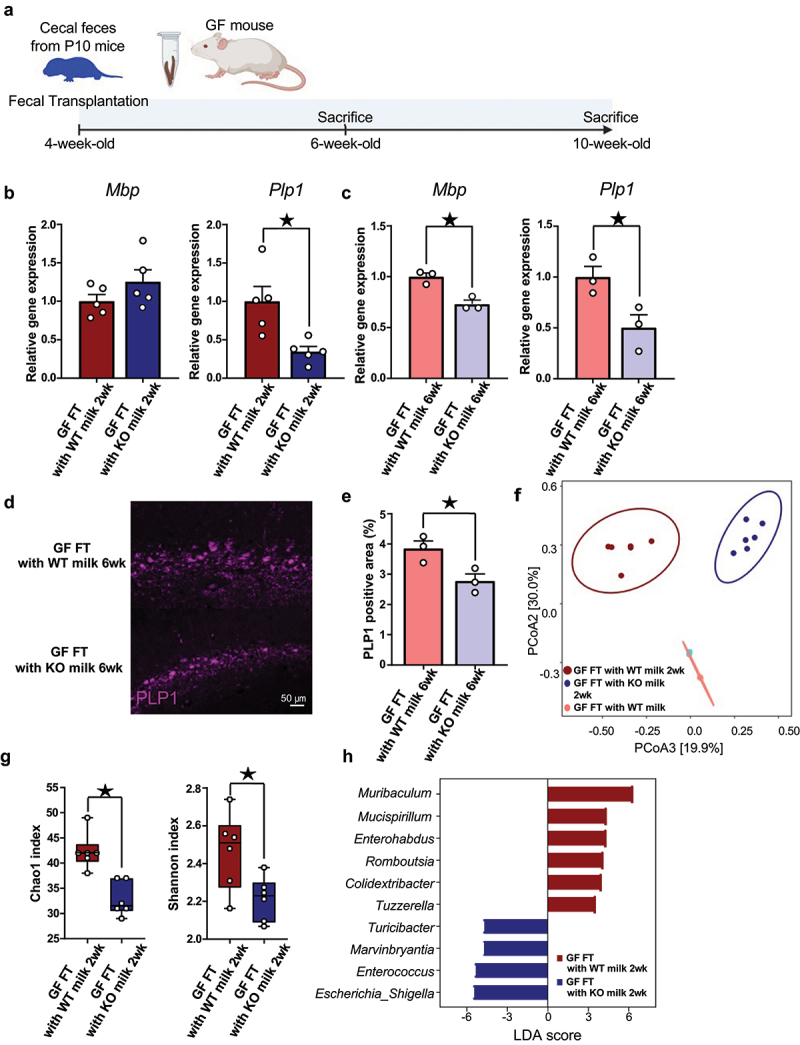
Fecal transplants from different breastfed neonates affect myelin-related gene expression in germ-free mice.

We conjectured that gut microbiome changes during infancy drive shifts in myelin-related gene expression and protein levels in the hippocampus of neonates due to recent studies have revealed a causal relationship between gut microbiome and myelin sheath integrity.^7–41^ Therefore, cecum feces from P10 *LAO1* KO with KO milk group and *LAO1* KO with WT milk group inoculated with the germ-free (GF) mice (Figure 3A). Gene expression analysis of samples collected at two weeks with fecal transplanted (FT) GF mice hippocampus demonstrated reduced *Plp1* expression in GF mice transplanted with feces from *LAO1* KO with KO milk neonates (referred to as GF FT with KO milk 2 wk). This aligned with our hypothesis when compared to GF mice transplanted with feces from *LAO1* KO with WT milk neonates (referred to as GF FT with WT milk 2 wk, Figure 3b). Furthermore, six weeks post-FT, hippocampal gene expression analysis revealed decreased expression of both *Plp1* and *Mbp* in GF FT with KO milk 6 wk (Figure 3c). This was corroborated by fluorescent immunostaining, which showed reduced PLP1 protein expression in the dorsal hippocampus (Figures 3d,e). Subsequent metagenomic analysis indicated different gut microbiome compositions between GF FT with WT milk 2 wk and GF FT with KO milk 2 wk, whereas gut microbiome compositions between GF FT with WT milk 6 wk and GF FT with KO milk 6 wk showed no change (Figure 3F). In addition, the richness and evenness of microbial communities were lower in GF FT with KO milk 2 wk compared to GF FT with WT milk 2 wk (Figure 3g). Further assessment by linear discriminant analysis (LDA) effect size (LEfSe) identified 10 differentially abundant taxa at genus level between GF FT with WT milk 2 wk and GF FT with KO milk 2 wk (Figure 3h). These findings suggested that LAO1-deficient breast milk changes the gut microbiome and influences the expression of hippocampal myelin-related genes and proteins. In addition, this gut microbiota-derived effect on hippocampal myelin was suggested to persist even when the gut microbiota composition was not maintained.

(a) Schematic overview illustrated the experiment design, in which GF mice were orally gavaged with cecum feces from P10 mice. Sampling was performed 2 and 6 weeks after fecal transplantation Created with BioRender.com.

(b) Quantitative real-time polymerase chain reaction (qRT-PCR) of *Mbp* and *Plp1* using hippocampus from 2 weeks after FT mice. *Mbp* (*n* = 5 each, *t*_*8*_ = 1.42, *p* = 0.19, Student’s *t*-test), *Plp1* (*n* = 5 each, *t*_*8*_ = 3.20, *p* < 0.05, Student’s *t*-test).

(c) QRT-PCR of *Mbp* and *Plp1* using hippocampus from 6 weeks after FT mice. *Mbp* (*n* = 3 each, *t*_*4*_ = 5.05, *p* < 0.05, Student’s *t*-test), *Plp1* (*n* = 3 each, *t*_*4*_ = 3.04, *p* < 0.05, Student’s *t*-test).

(d and e) Representative PLP1 expression at dorsal hippocampus from FT mice. Quantification of PLP1 positive area at dorsal hippocampus from FT mice. *n* = 3 each, *t*_*4*_ = 3.13, *p* < 0.05, Student’s *t*-test.

(f) Beta diversity of the 16S rRNA gene sequencing data sets from FT mice feces was determined using the Bray-Curtis index‘s principal coordinate analysis (PCoA). *n* = 6 each, *F* = 19.47, R-squared = 0.74, *p* < 0.05, PARMANOVA.

(g) Alpha-diversity was evaluated by chao1 and Shannon index. chao1 (*n* = 6 each, *U* = 0, *p* < 0.05, Mann-Whitney U test), Shannon (*n* = 6 each, *U* = 5, *p* < 0.05, Mann-Whitney U test)

(h) Histogram of genus-level taxa with p-values <0.05 evaluated by LEfSe analysis were visualized. *n* = 6 each.

Mean ± SEM; ^★^ indicates *p* < 0.05; GF, germ-free; FT, fecal transplantation; KO, knockout; WT, wild-type; 2 wk, two weeks after fecal transplantation; 6 wk, six weeks after fecal transplantation; LDA, linear discriminant analysis.

### Gut microbiome-derived metabolites contribute to hippocampal myelin-related gene expression

**Figure 4.**
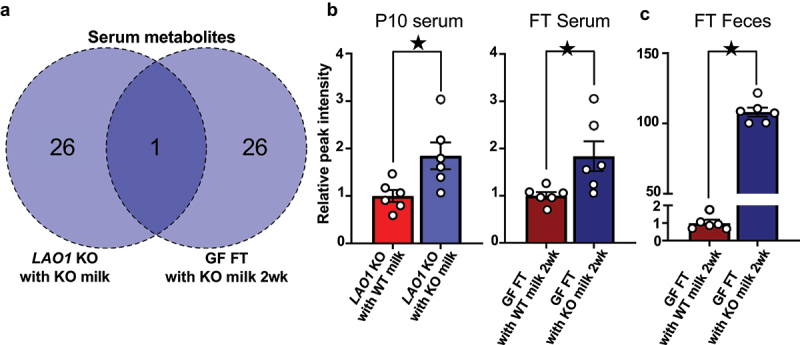
Gut microbiota influenced by LAO1 deficient augmented D-glucaric acid in serum and cecum feces.

We conducted non-targeted metabolomics on serum samples and compared the differences between *LAO1* KO with WT milk and *LAO1* KO with KO milk on P10 or between GF FT with WT milk 2 wk and GF FT with KO milk 2 wk. Serum metabolite profiles in both comparisons exhibited significant divergence based on naive-partial least square (PLS) analysis (Supplementary Figures S3A-S3D, and Supplementary Tables S2 and S3). In the search for metabolites showing common changes, D-glucaric acid was the only altered candidate metabolite in *LAO1* KO with KO milk on P10 and GF FT with KO milk 2 wk. (Figures 4a,b). Conversely, no shared alterations were found between sera from *LAO1* KO with WT milk on P10 and GF FT with WT milk 2 wk (Supplementary Figure S3E).

(a) Venn diagram of the P10 serum and FT serum metabolomics datasets from *LAO1* KO with KO milk-related samples constructed by naïve-partial least squares (PLS) analysis. Overlapped metabolite was D-glucaric acid. Blue indicated higher in *LAO1* KO with KO milk compared to LAO1 WT with KO milk. Dark blue indicated higher in GF FT with KO milk 2 wk compared to GF FT with WT milk 2 wk, and the numbers inside the Venn diagram indicate the metabolite counts that were elevated in each sample compared to the comparator.

(b) Relative peak intensity of D-glucaric acid from P10 serum, FT serum, and FT feces measured using gas chromatography-mass spectrometry (GC-MS) based metabolomics analysis. P10 serum (*n* = 6 each, *t*_*10*_ = 2,75, *p* < 0.05, Student’s *t*-test), FT serum (*n* = 6 each, *t*_*10*_ = 2.56, *p* < 0.05, Student’s *t*-test), FT feces (*n* = 6 each, *t*_*10*_ = 33.44, *p* < 0.05, Student’s *t*-test).

Mean ± SEM; ^★^ indicates *p* < 0.05; P, postnatal day; FT, fecal transplantation; KO, knockout; 2 wk, two weeks after fecal transplantation.

To investigate the D-glucaric acid origin in the serum, the glucaric acid quantity in feces after FT was compared and was confirmed to be higher in the GF FT with KO milk 2 wk than in the GF FT with WT milk 2 wk (Figure 4c). Further examination of fecal metabolites after FT revealed no change in D-glucuronic acid and D-glucuronolactone, precursors of D-glucaric acid; pyruvic acid, a metabolite of D-glucaric acid (Supplementary Figure S4 upper panel), increased. Microbiome-based prediction of enzyme expression showed that EC:1.2.1.3, which was the enzyme responsible for the conversion of D-glucuronolactone to D-glucaric acid, decreased in the feces of GF FT with KO milk 2 wk, whereas EC:4.2.1.40 and EC:4.1.2.20, which were involved in D-glucaric acid to pyruvic acid metabolism, increased (Supplementary Figure S4 bottom panel). Additionally, *Muribaculum* was found to have the greatest impact on the abundance of EC:1.2.1.3, whereas *Escherichia-Shigella* exhibited the highest influence on the abundance of EC:4.2.1.40 and EC:4.1.2.20 at genus level (Supplementary Tables S4–S6). The UniProt database^42,43^ indicated the absence of EC:4.2.1.40 and EC:4.1.2.20 in mammals, whereas mammals possess EC:1.2.1.3. In mice, the genes responsible for EC:1.2.1.3 are *Aldh1b1*, *Aldh2*, *Aldh3a2*, *Aldh7a1* and *Aldh9a1*. We compared gene expression using P10 *LAO1* KO with WT milk and *LAO1* KO with KO milk hepatic samples, which showed that breast milk difference didn’t influence hepatic EC:1.2.1.3 related gene expression (Supplementary Figure S5). The metabolite composition in WT and LAO1-deficient milk differed significantly; however, D-glucaric acid was not detected (Supplementary Figure S6). These results suggested that the altered gut microbiota composition of LAO1-deficient milk increased D-glucaric acid production, which might be transferred into the bloodstream.

### D-glucaric acid suppresses myelin-related gene expression in neonatal mice

**Figure 5.**
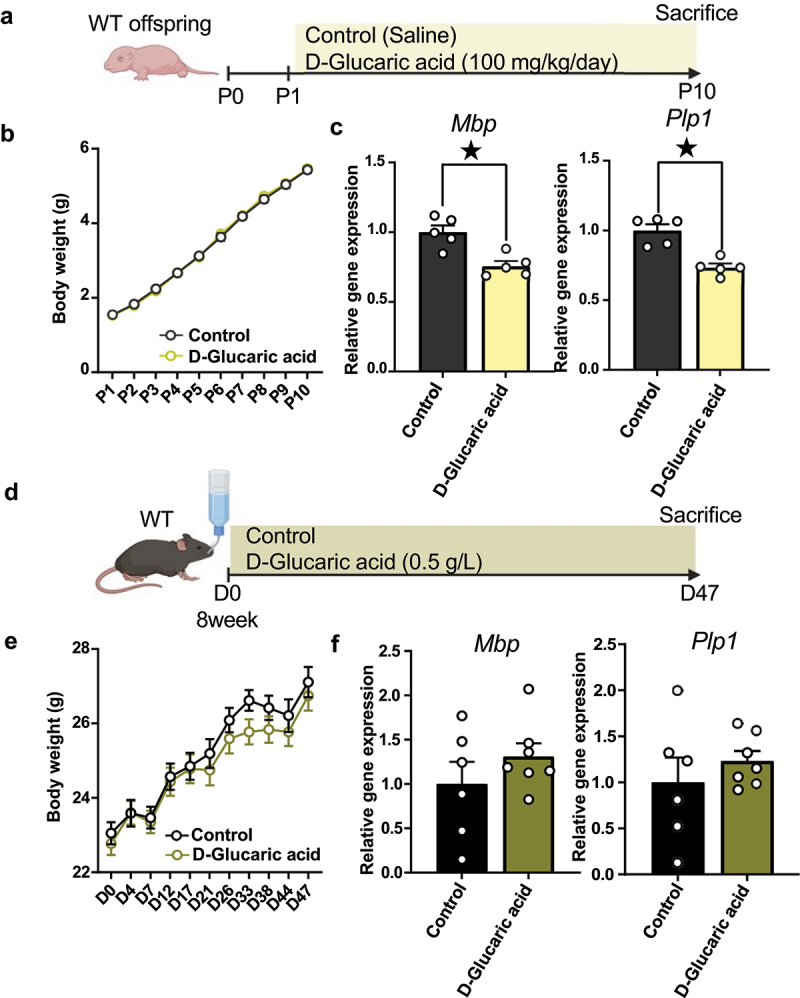
D-glucaric acid suppressed myelin-related gene expression in neonatal mice.

Considering common alterations in D-glucaric acid and its association with neurological disorders,^44,45^ we postulated that gut microbiome-derived D-glucaric acid could reduce hippocampal myelin-related gene expression. *In vivo* administration of D-glucaric acid to WT neonatal mice from P1 to P10 showed comparable body weight to controls, but the expression of hippocampal myelin-related genes (*Mbp, Plp1*) was significantly reduced at P10, echoing changes seen with distinct maternal milk compositions (Figures 5a–c). D-glucaric acid administration to WT adult mice for forty-seven days did not affect body weight and myelin-related gene expression (Figures 5d–f). To investigate the differences in absorption capacity between neonatal and adult, we measured tissue D-glucaric acid levels two hours after orally administration to both WT neonatal and adult mice. In infants, D-glucaric acid levels increased in the brain and liver, while didn’t change in adult (Supplementary Figures S7A and S7B). The outcome indicated that D-glucaric acid could thorough gut and blood-brain barrier during infancy but could not in adult. These findings suggest that excessive D-glucaric acid exposure during infancy suppresses myelin-related gene expression in the hippocampus.

(a) D-glucaric acid impact on neonatal hippocampal myelin-related gene was investigated, and the schematic diagram was illustrated. Created with BioRender.com.

(b) Body weight of P1–P10 100 mg/kg glucaric acid-treated mice. P1 (Control *n* = 18, D-Glucaric acid *n* = 19, *t*_*350*_ = 0.02, *p* > 0.99, Sidak’s tests), P2 (Control *n* = 18, D-Glucaric acid *n* = 19, *t*_*350*_ = 0.39, *p* > 0.99, Sidak’s tests), P3 (Control *n* = 18, D-Glucaric acid *n* = 19, *t*_*350*_ = 0.58, *p* > 0.99, Sidak’s tests), P4 (Control *n* = 18, D-Glucaric acid *n* = 19, *t*_*350*_ = 0.02, *p* > 0.99, Sidak’s tests), P5 (Control *n* = 18, D-Glucaric acid *n* = 19, *t*_*350*_ = 0.32, *p* > 0.99, Sidak’s tests), P6 (Control *n* = 18, D-Glucaric acid *n* = 19, *t*_*350*_ = 0.91, *p* = 0.99, Sidak’s tests), P7 (Control *n* = 18, D-Glucaric acid *n* = 19, *t*_*350*_ = 0.26, *p* > 0.99, Sidak’s tests), P8 (Control *n* = 18, D-Glucaric acid *n* = 19, *t*_*350*_ = 0.96, *p* = 0.98, Sidak’s tests), P9 (Control *n* = 18, D-Glucaric acid *n* = 19, *t*_*350*_ = 0.28, *p* > 0.99, Sidak’s tests), P10 (Control *n* = 18, D-Glucaric acid *n* = 19, *t*_*350*_ = 0.35, *p* > 0.99, Sidak’s tests).

(c) Quantitative real-time polymerase chain reaction (qRT-PCR) of *Mbp* and *Plp1* using hippocampus from P10 mice. *Mbp* (*n* = 5 each, *t*_*8*_ = 4.03, *p* < 0.05, Student’s *t*-test), *Plp1* (*n* = 5 each, *t*_*8*_ = 5.16, *p* < 0.05, Student’s *t*-test).

(d) D-glucaric acid impact on adult hippocampal myelin-related gene was investigated, and the schematic diagram was illustrated. Created with BioRender.com.

(e) Body weight of D1–D47 in glucaric acid-treated mice. D0 (Control *n* = 10, D-Glucaric acid *n* = 11, *t*_*19*_ = 0.65, *p* > 0.99, Sidak’s tests), D4 (Control *n* = 10, D-Glucaric acid *n* = 11, *t*_*19*_ = 0.02, *p* > 0.99, Sidak’s tests), D7 (Control *n* = 10, D-Glucaric acid *n* = 11, *t*_*19*_ = 0.27, *p* > 0.99, Sidak’s tests), D12 (Control *n* = 10, D-Glucaric acid *n* = 11, *t*_*19*_ = 0.26, *p* > 0.99, Sidak’s tests), D17 (Control *n* = 10, D-Glucaric acid *n* = 11, *t*_*19*_ = 0.13, *p* > 0.99, Sidak’s tests), D21 (Control *n* = 10, D-Glucaric acid *n* = 11, *t*_*19*_ = 0.79, *p* = 0.99, Sidak’s tests), D26 (Control *n* = 10, D-Glucaric acid *n* = 11, *t*_*19*_ = 0.97, *p* = 0.99, Sidak’s tests), D33 (Control *n* = 10, D-Glucaric acid *n* = 11, *t*_*19*_ = 1.94, *p* = 0.54, Sidak’s tests), D38 (Control *n* = 10, D-Glucaric acid *n* = 11, *t*_*19*_ = 1.21, *p* = 0.95, Sidak’s tests), D44 (Control *n* = 10, D-Glucaric acid *n* = 11, *t*_*19*_ = 0.75, *p* > 0.99, Sidak’s tests), D47 (Control *n* = 10, D-Glucaric acid *n* = 11, *t*_*19*_ = 0.64, *p* > 0.99, Sidak’s tests).

(f) qRT-PCR of *Mbp* and *Plp1* using hippocampus from treated adult mice. *Mbp* (Control *n* = 6, D-Glucaric acid *n* = 7, *t*_*11*_ = 0.29, *p* = 0.29, Student’s *t*-test), *Plp1* (Control *n* = 6, D-Glucaric acid *n* = 7, *t*_*11*_ = 0.85, *p* = 0.41, Student’s *t*-test).

Mean ± SEM; ^★^ indicates *p* < 0.05; P, postnatal day; D, day.

**Figure 6.**
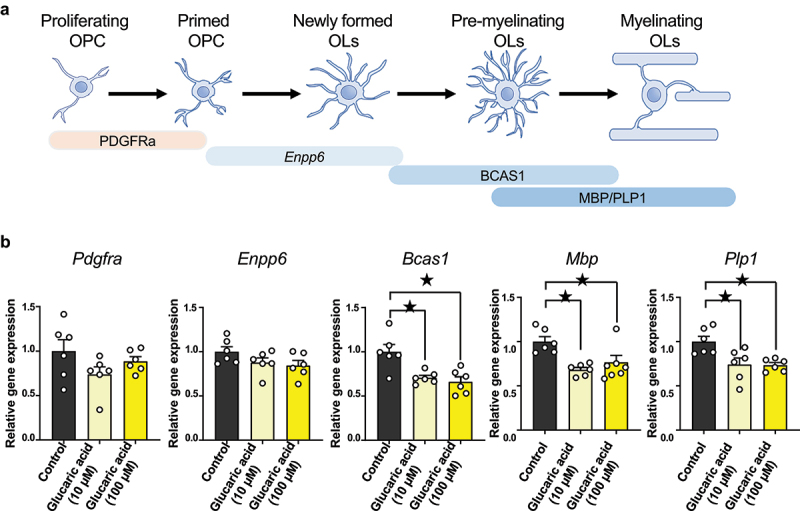
D-glucaric acid treatment induces gene expression change in OPC differentiation-conditioned cells.

*In vitro* studies were also conducted to determine the possibility that this effect was directly caused by D-glucaric acid and to seek the potential molecular mechanism of it. As shown in Figure 6a, it is known that *Pdgfra*, *Enpp6*, *Bcas1*, and *Mbp*/*Plp1* are sequentially expressed from OPCs to myelinating OLs.^46^ We cultured primary OPCs under differentiation conditions with D-glucaric acid and examined the expression of these genes. D-glucaric acid treatment decreased *Bcas1* and *Mbp*/*Plp1* expression, but *Pdgfra* and *Enpp6* expression remained unaffected (Figure 6b). These results suggest that D-glucaric acid might inhibit pre-myelinating oligodendrocyte development during infancy.

(a) Representative OPC to myelinating OL marker. *Pdgfra* is a progenitor marker. *Enpp6* is the pre- and immature OL marker. *Bcas1* represents pre- and immature OL to myelinating OL marker. *Mbp* and *Plp1* are myelinating OL markers.

(b) Quantitative real-time polymerase chain reaction (qRT-PCR) of *Pdgfra*, *Enpp6, Bcas1, Mbp*, and *Plp1* from OPC differentiation-conditioned cells. *Pdgfra* (*n* = 6 each, Control versus Glucaric acid 10 μM: *q*_*15*_ = 1.99, *p* = 0.11, Control versus Glucaric acid 100 μM: *q*_*15*_ = 0.86, *p* = 0.61, Dunnett’s test), *Enpp6* (*n* = 6 each, Control versus Glucaric acid 10 μM: *q*_*15*_ = 1.57, *p* = 0.23, Control versus Glucaric acid 100 μM: *q*_*15*_ = 0.20, *p* = 0.11, Dunnett’s test), *Bcas1* (*n* = 6 each, Control versus Glucaric acid 10 μM: *q*_*15*_ = 3.50, *p* < 0.05, Control versus Glucaric acid 100 μM: *q*_*15*_ = 4.04, *p* < 0.05, Dunnett’s test), *Mbp* (*n* = 6 each, Control versus Glucaric acid 10 μM: *q*_*15*_ = 3.92, *p* < 0.05, Control versus Glucaric acid 100 μM: *q*_*15*_ = 2.87, *p* < 0.05, Dunnett’s test), *Plp1* (*n* = 6 each, Control versus Glucaric acid 10 μM: *q*_*15*_ = 3.21, *p* < 0.05, Control versus Glucaric acid 100 μM: *q*_*15*_ = 3.30, *p* < 0.05, Dunnett’s test).

Mean ± SEM; ^★^ indicates *p* < 0.05; OPC, oligodendrocyte precursor cells; OL, oligodendrocytes.

### H_2_O_2_ ameliorates myelin-related gene expression and D-glucaric acid production in neonatal mice

**Figure 7.**
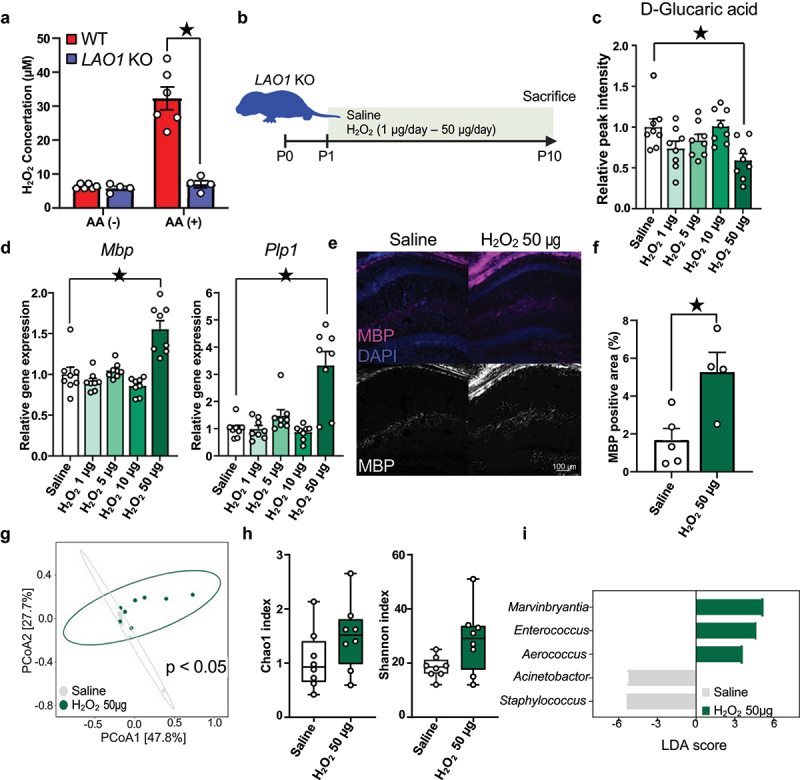
H_2_O_2_ ameliorates myelin-related gene expression and D-glucaric acid production in neonatal mice.

LAO1 in breast milk catalyzes the production of L-amino acids and generates H_2_O_2_ (Figure 7a). Taking into consideration the catalytic and antibacterial properties of H_2_O_2_,^47–49^ we predicted that a decrease in H_2_O_2_ concentration in milk due to LAO1 deficiency would be associated with gut microbiota disturbance and increased D-glucaric acid production. Administration of varying H_2_O_2_ doses to *LAO1* KO neonates from P1 to P10 revealed decreased D-glucaric acid serum levels in the H_2_O_2_ 50 μg group (Figures 7b,c).

(a) H_2_O_2_ concentration after incubating skim milk with or without amino acids (AA). AA (-) (WT milk *n* = 6, *LAO1* KO milk *n* = 4, *t*_*8*_ = 0.83, *p* = 0.43, Student’s *t*-test.), AA (+) (WT milk *n* = 6, *LAO1* KO milk *n* = 4, *t*_*8*_ = 5.89, *p* < 0.05, Student’s *t*-test).

(b) Investigated the impact of orally administrated H_2_O_2_ to neonatal *LAO1* KO mice, and the schematic diagram was illustrated. Created with BioRender.com.

(c) Relative peak intensity of D-glucaric acid from serum metabolomics. *n* = 8 each, Saline versus H_2_O_2_ 1 μg/day: *q*_*35*_ = 2.167, *p* = 0.12, Saline versus H_2_O_2_ 5 μg/day: *q*_*35*_ = 1.35, *p* = 0.47, Saline versus H_2_O_2_ 10 μg/day: *q*_*35*_ = 0.07, *p* = 0.99, Saline versus H_2_O_2_ 50 μg/day: *q*_*35*_ = 3.36, *p* < 0.05, Dunnett’s test.

(d) Quantitative real-time polymerase chain reaction (qRT-PCR) of *Mbp* and *Plp1* using the hippocampus of H_2_O_2_-supplemented 10-day-old neonates. *Mbp* (*n* = 8 each, Saline versus H_2_O_2_ 1 μg/day: *q*_*35*_ = 1.533, *p* = 0.36, Saline versus H_2_O_2_ 5 μg/day: *q*_*35*_ = 0.1739, *p* = 0.99, Saline versus H_2_O_2_ 10 μg/day: *q*_*35*_ = 2.21, *p* = 0.11, Saline versus H_2_O_2_ 50 μg/day: *q*_*35*_ = 6.006, *p* < 0.05, Dunnett’s test), *Plp1* (*n* = 8 each, Saline versus H_2_O_2_ 1 μg/day: *q*_*35*_ = 0.4061, *p* = 0.98, Saline versus H_2_O_2_ 5 μg/day: *q*_*35*_ = 1.061, *p* = 0.67, Saline versus H_2_O_2_ 10 μg/day: *q*_*35*_ = 0.7489, *p* = 0.87, Saline versus H_2_O_2_ 50 μg/day: *q*_*35*_ = 7.767, *p* < 0.05, Dunnett’s test).

(e and f) Representative myelin basic protein (MBP) staining and quantitative analysis of P10 *LAO1* KO mice dorsal hippocampus. Saline *n* = 5, H_2_O_2_ 50 μg/day *n* = 4, *t*_*7*_ = 3.14, *p* < 0.05, Student’s *t*-test.

(g) Beta diversity of the 16S rRNA gene sequencing data sets from Saline and H_2_O_2_ 50 μg/day-administrated *LAO1* KO neonate feces as determined using principal coordinate analysis (PCoA) of Bray-Curtis index. *n* = 8 each, *F* = 2.21, R-squared = 0.14, *p* < 0.05, PERMANOVA.

(h) Alpha-diversity was evaluated by chao1 and Shannon index. chao1 (*n* = 8 each, *U* = 15, *p* = 0.08, Mann-Whitney U test), Shannon (*n* = 8 each, *U* = 19, *p* = 0.19, Mann-Whitney U test).

(i) Histogram of genus-level taxa with p-values <0.05 evaluated by LEfSe analysis were visualized (*n* = 8 each).

Mean ± SEM; ^★^ indicates *p* < 0.05; KO, knockout; WT, wild-type.

Furthermore, the H_2_O_2_ 50 μg group showed increased hippocampal myelin-related gene expression (*Mbp* and *Plp1*) compared to saline controls (Figure 7d), supported by higher MBP protein levels in the dorsal hippocampus (Figures 7e,f). A 16s rRNA metagenome analysis exhibited differential gut microbiome communities between saline and H_2_O_2_ 50 μg groups (Figure 7g). Daily administrating H_2_O_2_ 50 μg didn’t influence on the richness and evenness of microbial communities (Figure 7h). Linear discriminant analysis (LDA) effect size (LEfSe) found the abundance of five taxa at genus level was altered (Figure 7i). On the other hands, H_2_O_2_ 50 μg group didn’t show upregulation of representative antioxidant genes expression in ileum and rectum (Supplementary Figures S8 and S9). These findings suggest that oral administration of H_2_O_2_ modulates the gut microbiome and decreases D-glucaric acid production, critically affecting brain development, particularly myelination, via infancy-associated blood metabolites.

## 3. Discussion

Recently, the microbiota-gut-brain axis has been increasingly researched, and current findings acknowledge the potential role of breast milk in gut microbiota formation and brain development. Previous studies have primarily examined the effects of breast milk and gut microbiome on the brain in isolation. Breast milk intake directly influences brain development and contributes to the gut microbiome composition. Therefore, to explore mechanisms of the microbiota-gut-brain axis on brain development during infancy, considering the gut microbiota influence established by breast milk is essential; suitable animal models have been sought to investigate it. We previously found that mouse milk contained one enzyme, LAO1, which could generate H_2_O_2_ and was involved in antibacterial action on the mother’s mammary glands and offspring gut microbiota formation.^13,15^ Here, we provide the first report that LAO1 in the mother’s milk is vital for proper gut microbiota formation and brain development in the offspring. In particular, gut microbiota changes due to decreased milk H_2_O_2_ levels in *LAO1*-deficient mice may result in abnormal D-glucaric acid synthesis, and the D-glucaric acid transferred into the circulation may inhibit myelin-related gene expression in the offspring hippocampus, thereby affecting mouse brain function after growth.

Brain development is particularly vulnerable to the adverse effects of early life dysbiosis.^50,51^ In this study, our results demonstrated that early life dysbiosis induced by insufficient H_2_O_2_ levels of LAO1-absent breast milk impaired spatial learning in adults‘ Morris water maze test and object location test. A previous study also indicated that early life dysbiosis caused by antibiotic treatment from P1 to P28 induced a disability in spatial learning and memory in this test.^52^ In addition, antibiotic treatment of juvenile (P21–P100) mice resulted in spatial learning impairment in the test.^53^ Antibiotic administration only infantile period (P4–P13) has not been reported to impair spatial memory learning in the Morris water maze test,^54^ possibly because of the maternal effect such as maternal microbiome and breast milk preserve infant microbiome formation from antibiotics disturbance. Increasing evidence has shown that various factors are involved in early life dysbiosis, including delivery mode, breastfeeding, and antibiotic administration.^55^ The effects of dysbiosis during early life on spatial memory after adulthood from multiple angles using various models must be investigated; our evidence suggests a strong relationship between spatial memory impairment and early life dysbiosis.

We propose that the decreased myelin gene and protein expression during infancy is associated with spatial memory deficits in adulthood. Axon myelination has recently shown to be essential for increasing conduction velocity of long-distance axons and brain functions, such as spatial memory.^46–59^ Essential evidence for OL and myelin requirement in spatial learning has been investigated in recent years. Steadman et al. showed that inhibiting OPC differentiation disturbed the recall of spatial memory but not initial learning in the Morris water maze test in adult mice.^60^ Wang et al. demonstrated that impairing OPCs disturbed recall of spatial memory but not initial learning in adult mice and increased OPC differentiation improved recall without changing initial learning in aged mice.^61^ Another study by Chen et al. also indicated that enhancing myelin renewal genetically (M1R cKO) or pharmacologically (Clemastine) in Alzheimer’s mouse model improved recall without initial learning changes in adult mice.^62^ These studies indicate that OPC differentiation is essential for recall, but not for initial learning, in adult mice. Myelin mostly develops in the early postnatal period^63^; however, the effects of OPC differentiation in infancy and myelin on adult spatial memory remain unelucidated. A previous study reported that mice exposed to 1.5% isoflurane for 4 h on postnatal day 7 showed impaired spatial memory in the novel object location and Y-maze tests in adults with abnormal oligodendrocyte development in juveniles; pharmacologically (Clemastine) enhancing oligodendrocyte differentiation ameliorated spatial memory.^64^ Another study reported that maternal sevoflurane exposure caused initial spatial learning and recall deficits in the adult offspring of mice that lost hippocampal myelin as neonatals.^65^ These studies imply that early life adverse treatment with OLs can induce behavioral abnormalities, such as spatial memory. In contrast, early life exposure to isoflurane or sevoflurane has shown broad influences on the central nervous system, such as astrogliosis^66^ and reduced neurogenessis.^67^ Therefore, the causal relationship between myelin impairment in neonates and spatial memory in adults needs to be proven using genetic and/or pharmacological approaches.

Our results showed that the reduced myelin gene expression was caused by elevated D-glucaric acid levels in the intestinal tract and blood. D-glucaric acid is derived from D-glucose and has several positive effects, such as cancer initiation inhibition^68^ and serum cholesterol reduction.^69^ Conversely, blood from patients with autism^44^ and postmortem brains from patients with bipolar patient,^45^ respectively, show high D-glucaric acid levels. Neurodevelopmental disorders such as ASD^70^ and bipolar disorder^56^ present with abnormal myelin levels. Thus, our study may demonstrate a mechanism of myelin depletion in these disorders. On the other hand, the administration of D-glucaric acid did not reduce myelin gene levels to the extent observed in *LAO1* KO mice. This may have been due to inadequate doses of D-glucaric acid; hence, further studies are needed considering dosage, administration route, and duration. We also demonstrated that D-glucaric acid directly inhibited oligodendrocyte gene expression. OPCs differentiation *in vitro* showed that *Bcas1* and *Mbp*/*Plp1* expressions were inhibited by D-glucaric acid treatment, but *Pdgfra* and *Enpp6* expressions were not inhibited. These results suggest that D-glucaric acid may affect the pre-myelinating step in OLs development in neonatal mice.

We also showed a particular window during which D-glucaric acid acted on hippocampal myelin gene expression. D-glucaric acid administration to neonatal mice inhibited *Mbp* and *Plp1* expression in the hippocampus, whereas administration to 8-week-old mice did not show alteration. This result indicates that mice already have mature barrier function from eight weeks, whereas neonatal barrier function is still in progress. Our results in Supplementary Figure S7 and some evidence supports this idea; first, the intestinal barrier is weaker in neonates compared to adults.^71^ Second, the blood-brain barrier susceptibility is higher in neonates than in adults ,^72–74^ Thus, D-glucaric acid could reach the CNS during infancy when the barrier is immature but not after growth when the barrier function is complete.

In this study, we focused on myelination and oligodendrocyte development, which were enriched in *LAO1* KO with WT milk based on microarray analysis and Gene Ontology (GO) analysis. The results revealed that the LAO1 deficiency in breast milk affects the development of myelin-related genes during infancy via alteration of the gut microbiota. Conversely, several GO terms, such as which were negative regulation of cell differentiation and G protein-coupled receptor activity, enriched in *LAO1* KO with KO milk. The elevated levels of D-glucaric acid were found to inhibit oligodendrocyte differentiation. Thus, focusing on the negative regulation of cell differentiation, key factors such as *Delta-Like Protein 1* (*Dll1*) and *octamer-binding transcription factor 4* (*Oct-4*) were found to be reduced. *Dll1* is known as a ligand for the Notch signaling pathway.^75^ Inhibition of Notch signaling pathway has been previously recognized to inhibit oligodendrocyte differentiation.^76,77^ Oct-4 widely expresses stem cells, although the detailed effect of Oct-4 on OPCs and OLs remains unclear, introducing *Oct-4* into in vitro OPCs has been reported to promote differentiation.^78,79^ The effect of D-glucaric acid might be associated with the downregulation of *Dll1* and/or *Oct-4* expression, leading to a decrease in the expression of myelin-related genes. However, the mechanisms by which D-glucaric acid directly affected pre-myelinating OLs differentiation is still elusive. Future in vitro experiments for identifying detail molecular mechanism, including receptor and pathway, of D-glucaric acid is needed to make clear how this metabolite inhibits pre-myelinating OLs differentiation. This approach may provide insights into the intricate pathways through which LAO1-deficient breast milk-induced alterations in the gut microbiota and D-glucaric acid impact the expression of key regulators such as *Dll1* and *Oct-4*.

We provide a novel mechanism by which the gut microbiota, regulated via H_2_O_2_ exposure during infancy, improves myelin-related gene expression by reducing D-glucaric acid levels. We are convinced that excess H_2_O_2_ is harmful; however, H2O2 is utilized via redox activity for various physiological functions, including cell signaling^80^ and antimicrobial activity.^48,49^ Hydrogen oxygen has been reported in breast milk derived from xanthine oxidase is used for antimicrobial activity and that ROS-producing enzymes regulate the gut microbiota.^81,82^ Our study and other studies also show that gut bacteria have a different tolerance to H_2_O_2_.^14,15^ Thus, H_2_O_2_ production derived from enzymes in breast milk may be a gatekeeper in gut microbiota formation during infancy, inhibiting the production of harmful metabolites such as D-glucaric acid and positively affecting host brain development. As this study did not identify bacteria directly associated with D-glucaric acid production, however; we suggested that *Muribaculum* and *Escherichia-Shigella* might have a connection based on predicting metagenomics. Previous studies reported that *Muribaculum* administration inhibited infectious enteritis^83^ and pharmacologically induced enteritis.^84^ Regarding *Escherichia-Shigella*, this taxon was shown associated with tumor necrosis factor alpha level in blood and blood-brain barrier permeability.^85^ Based on these results, *Muribaculum* might positively influence on host while *Escherichia-Shigella* might be as pathobiont. However, our data indicated there were a lot of reads which were not assigned to the genus, and we only have shown the predicted result (Supplementary Tables S4–S6). Further investigation using bacterial culture and/or long-read sequencing may be helpful in the future to identify the direct association individual taxa with D-glucaric acid production.

LAO1 catabolites L-amino acids and produces keto acids, ammonia, and H_2_O_2_. In this study, we focused on the function of H_2_O_2_ in the neonate’s gut microbiota formation, metabolite change, and brain development. On the other hand, metabolomics analysis identified that LAO1-deficient breast milk increased not only L-amino acids but also decreased keto acids such as phenylpyruvic acid and oxoglutaric acid. Oxoglutaric acid, which is also known as alpha-ketoglutaric acid, is derived from L-glutamic acid, and this metabolite is intermediate to the citrate cycle; thus, reducing this metabolite might induce inefficient energy production.^86^ Phenylpyruvic acid is derived from L-phenyl alanine. Previous studies reported that administrating phenylpyruvic acid improves growth in rodents and chickens.^87,88^ In addition, it has been proposed that keto acids, which are phenylpyruvic acid, indole-3-pyruvic aid, and 4-hydroxyphenylpyruvic acid, may play a crucial role in the post-injury remyelination of the nervous system.^89^ In fact, detailed investigations into the actual impact of these keto acids on brain development and the formation of the gut microbiota have not yet been conducted. However, considering the findings from previous studies and the results of our research, it is plausible to suggest that the metabolites produced by LAO1, in addition to serving as energy sources, may also exert physiological effects as substances supporting brain development, specifically myelin development, and influencing the composition of the gut microbiota. Conducting research using *LAO1* KO mice in the future could be beneficial in understanding how keto acids effecting on the development of offspring.

In conclusion, we comprehensively and objectively demonstrated that breast milk components were involved in brain development during infancy via forming gut microbiota in a mouse model. In particular, LAO1 activity in breast milk for normal gut microbiota formation and the adverse effects of D-glucaric acid on brain development in neonates should be further investigated in humans and other animals. In addition, the association of this finding with ASD, ADHD, and anxiety will also be examined, which may lead to the development of new therapeutic agents.

## Supplementary Material

Supplementary data set1.xlsx

## Data Availability

The microarray data and metagenomics data were deposited in GSE246024, PRJNA1029238 and PRJNA1029237. Any additional information will be shared upon request.
