# Correction

**DOI:** 10.1080/19490976.2024.2377878

**Published:** 2024-07-19

**Article title**: Hydrogen peroxide in breast milk is crucial for gut microbiota formation and myelin development in neonatal mice

**Authors**: Kambe J, Usuda K, Inoue R, Hirayama K, Ito M, Suenaga K, Masukado S, Liu H, Miyata S, Li C, Kimura I, Yamamoto Y, and Nagaoka K.

**Journal**: *Gut Microbes*

**DOI**: https://doi.org/10.1080/19490976.2024.2359729

The article was originally published without Graphical Abstract.

Graphical Abstract provided below have been included in the original article, and it has been republished accordingly.

**Figure:**
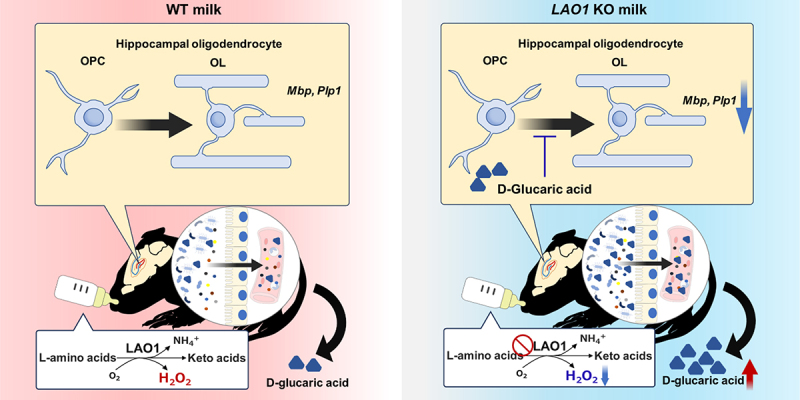

The article was originally published without Supplementary materials. Supplementary materials have been uploaded and republished accordingly.

